# Profound Climatic Effects on Two East Asian Black-Throated Tits (Ave: Aegithalidae), Revealed by Ecological Niche Models and Phylogeographic Analysis

**DOI:** 10.1371/journal.pone.0029329

**Published:** 2011-12-16

**Authors:** Chuanyin Dai, Na Zhao, Wenjuan Wang, Congtian Lin, Bin Gao, Xiaojun Yang, Zhengwang Zhang, Fumin Lei

**Affiliations:** 1 Key Laboratory of Zoological Systematics and Evolution, Institute of Zoology, Chinese Academy of Sciences, Beijing, China; 2 State Key Laboratory of Genetic Resources and Evolution, Kunming Institute of Zoology, Chinese Academy of Sciences, Kunming, China; 3 Ministry of Education Key Laboratory for Biodiversity Science and Ecological Engineering, College of Life Sciences, Beijing Normal University, Beijing, China; 4 Graduate University of Chinese Academy of Sciences, Beijing, China; Smithsonian Institution National Zoological Park, United States of America

## Abstract

Although a number of studies have assessed the effects of geological and climatic changes on species distributions in East Asian, we still have limited knowledge of how these changes have impacted avian species in south-western and southern China. Here, we aim to study paleo-climatic effects on an East Asian bird, two subspecies of black-throated tit (*A. c. talifuensis–concinnus*) with the combined analysis of phylogeography and Ecological Niche Models (ENMs). We sequenced three mitochondrial DNA markers from 32 populations (203 individuals) and used phylogenetic inferences to reconstruct the intra-specific relationships among haplotypes. Population genetic analyses were undertaken to gain insight into the demographic history of these populations. We used ENMs to predict the distribution of target species during three periods; last inter-glacial (LIG), last glacial maximum (LGM) and present. We found three highly supported, monophyletic MtDNA lineages and different historical demography among lineages in *A. c. talifuensis–concinnus*. These lineages formed a narrowly circumscribed intra-specific contact zone. The estimated times of lineage divergences were about 2.4 Ma and 0.32 Ma respectively. ENMs predictions were similar between present and LGM but substantially reduced during LIG. ENMs reconstructions and molecular dating suggest that Pleistocene climate changes had triggered and shaped the genetic structure of black-throated tit. Interestingly, in contrast to profound impacts of other glacial cycles, ENMs and phylogeographic analysis suggest that LGM had limited effect on these two subspecies. ENMs also suggest that Pleistocene climatic oscillations enabled the formation of the contact zone and thus support the refuge theory.

## Introduction

Phylogeographic analysis is a powerful way of gaining insights into the historical processes that have shaped current species distribution and genetic variation [1]. Combined analysis of geographical and genealogical information has revealed that Pleistocene glacial cycles and climate changes had profound impacts on species' distribution patterns and population diversity [2]–[5]. For instance, species inhabiting high-latitude regions of Europe and North America, which had been covered by the heavy ice sheets usually have shallow genetic divergences and low genetic diversity, whereas species living in the temperate refugial areas of these regions generally have deep divergences and higher genetic diversity [6]–[9]. Ecological data can complement phylogeographic research by providing information on species' origin, evolutionary history and present distribution. One way to use these ecological data is in ecological niche models (ENMs), which use collection localities and Geographic Information System (GIS) maps of ecological data to develop spatial predictions of a species' historical and current range [10]–[11]. This approach has been somewhat controversial because it assumes that information on the distribution of ancestral species at the time of speciation can be deduced from the current distribution of their descendent species [12]–[14]. Nonetheless, integrating phylogeographic and ecological analysis has become more common in recent years, and this approach has provided new insights on speciation and species' distribution [15]–[17].

South-western and southern China is one of the most species-rich regions in the world. This region ranges from an elevation of more than 2000 m on the Yunnan-Guizhou (Yungui) plateau to the medium and low mountains (lower than 1800 m) areas of East China. Many theories have been proposed to explain its extraordinary number of species. For instance, this area may have served as a refugium during the Pleistocene glaciations [18] or as the center of speciation [19]. Zhang [20] suggested that this region was not glaciated throughout the Quaternary period. However, even in the absence of ice sheets, the habitats in this region are believed to have been much affected by the climatic oscillations as well as the uplift of the Qinghai-Tibet plateau [21]. These major climatic and geographical events are likely to have profoundly affected the distributions, and consequently the population dynamics, of many species. Previous studies on the avifauna of this region have highlighted genetic differentiation, unusual historical demography and the existence of multiple refugia during the glacial periods [22]–[25]. Although these studies have assessed the effects of geological and climatic changes on species distributions in East Asian, we still have limited knowledge of how these changes have impacted avian species in south-western and southern China. Improving our understanding of this is important for estimating the global effects of Pleistocene climate variations, for predicting future climate change impacts and for setting priorities for conservation and management [26]–[27]. Obviously, such questions can be best addressed by integrating phylogeographic analysis with ecological niche models because these two kinds of analysis are independent of each other and thus offer independent information.

The black-throated tit *(Aegithalos concinnus)* is a wide-spread and small passerine bird that lives in pine and open broadleaf forest, generally at middle altitudes. Its range extends from the foothills of the Himalayas in Pakistan and northern India through large areas of southern China to northern Burma, Vietnam and Taiwan. Currently, six subspecies are recognized; *A. c. concinnus, A. c. talifuensis, A. c. iredalei, A. c. manipurensis, A. c. pulchellus* and *A. c. annamensis*, some of which differ markedly in plumage pattern and coloration. For instance, *A. c. pulchellus* and *A. c. annamensis* are gray-headed but the other subspecies are red-headed. Studies have discovered significant genetic divergence among these red crowned subspecies [28]–[29]. Genetic distances between groups range from 4.3% within the subspecies *A. c. iredalei* to 5.4–5.8% between *A. c. iredalei* and *A. c. manipurensis*, and up to 5.3–5.8% between the latter two subspecies and subspecies *A. c. talifuensis*–*concinnus* [29]. Although the phylogeny of the *A. concinnus* complex has not been resolved, the monophyly of *A. c. talifuensis–concinnus* was strongly supported by the nuclear and MtDNA makers [29]. These two subspecies occupy the largest part of the species' distribution range, being mainly distributed in south-western and southern China. Races in this group have similar morphology except that the coloration of the crown and chestnut breast band is redder in the nominate subspecies *(A. c. concinnus).* However, in our previous survey of the phylogenetic relationships of the long-tailed tits, we discovered substantial genetic differentiation between four black-throated tit specimens, including two *A. c. concinnus* and two *A. c. talifuensis* [30].

In this paper, we aim to improve the understanding of glacial impacts on East Asian birds by coupling phylogeographic analysis and ecological niche models to investigate the population structure and demography of the intensive sampling specimens of *A. c. talifuensis–concinnus*. MtDNA sequences served as our genetic data because its patterns can be robust indicators of patterns of population [31]–[32]. We will specifically attempt to:

1. Describe the geographic structure of genetic variation within *A. c. talifuensis–concinnus* and the historical demography of any identified lineages.
2. Illustrate the effects of climate changes on the genetic divergences of *A. c. talifuensis–concinnus*.
3. Determine if last glacial maximum (LGM) had an impact on this species.
4. Identify the mechanism for the formation of the narrowly circumscribed contact zone.

## Methods

### Ethics statement

All used samples are unprotected bird specimens from the specimen collection of the National Zoological Museum, Institute of Zoology, Chinese Academy of Sciences (address: No1 Beichen West Road, Chaoyang District, Beijing, China). The collection was under the permit from Forestry Department and conformed to the National Wildlife Conservation Law in China. No living animal experiments were conducted in the current research.

### Sample collection, DNA extraction, PCR amplification and sequencing

**Figure 1:**
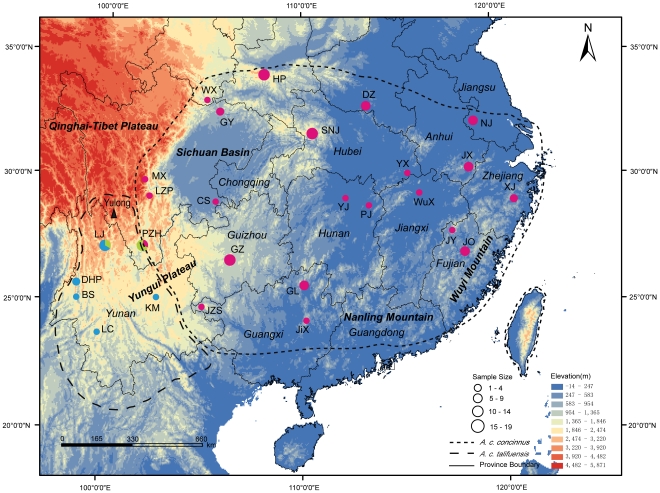
Sampling sites, geographical and lineages distribution of *A. c. talifuensis–concinnus*. Circles and associated letters indicate sample sizes and sampling localities. Each color represents a lineage. PZH includes populations in Panzhihua, Miyi, Yanbian and Yanyuan Counties and LC includes populations in Gengma, Shuangjiang and Yunxian Counties. The distribution of subspecies is based on that in Chinese Fauna Sinica [80], but has been slightly modified based on our field observations.

We examined a total of 203 individuals from 32 localities within the breeding range of *A. c. talifuensis–concinnus* (Fig. 1). Total genomic DNA was extracted from blood or tissue samples using the QIAGEN DNeasy Tissue kit following the manufacturer's instructions. Polymerase chain reaction (PCR) was used to amplify the Cytochrome c oxidase subunit I (COI), Cytochrome b (*cyt b*) and NADH dehydrogenase subunit 2 (ND2) genes. As described in [33], the primers H7956 and L6615 were used to amplify COI, and H6313 and L5219 for ND2. PCR reactions were run using the following parameters: denaturation at 94°C for 2 min, followed by 40 cycles at 93°C for 1 min, 46°C for COI and 53°C for ND2 for 45 s, and 72°C for 2 min, and a final 8 min at 72°C. The primers and amplification conditions used for cytochrome b were as described in [34].

Each round of PCR reactions also included one negative control to check for contamination. The PCR products were purified using a QIAquick PCR purification kit (QIAGEN) and sequenced on a Perkin-Elmer 377 semiautomated DNA sequencer (Applied BioSystems) using a Perkin-Elmer Prism terminator cycle sequencing kit (Applied BioSystems) with Ampli Taq FS polymerase and BigDye terminators. Both strands of each PCR product were sequenced. Complete sequences were assembled using Seqman II (DNASTAR). Sequences were compared visually to the original chromatograms to avoid reading errors. All sequences are accessible at GenBank under the accession No. HQ605086 - HQ605288 for COI, No. HQ605289 - HQ605491 for *cyt b* and No. HQ605492 - HQ605694 for ND2.

### Genetic diversity

Sequences for COI, *cyt b* and ND2 were aligned using ClustalW as implemented in MEGA 4 [35]. Since all fragments belong to the same DNA molecule, all subsequent analyses were performed with three sequences per individual concatenated in three partitions: COI from nucleotide 1–1227, *cyt b* from nucleotide 1228–2211 and ND2 from nucleotide 2212–3192. The number of segregating sites, haplotype diversity and nucleotide diversity for each population and all populations combined were estimated using DnaSP 5.0 [36].

### Phylogenetic analyses

Maximum likelihood (ML) and Bayesian methods (BI) were used to identify major lineages and evaluate relationships among haplotypes. An individual from the great tit *(Parus major)* and long-tailed tit *(Aegithalos caudatus)* were used as outgroups. The substitution models for each of the three gene sequence and the concatenated data sets were selected using MrMODELTEST 2.2 [37] as implemented in MrMTgui (http://genedrift.org/mtgui.php) based on the Akaike information criterion [38]. The best fit models for the COI and ND2 sequence data sets were GTR+I. The best fit model for the cytochrome-b sequence data set was HKY+I and the GTR+I+G model was selected as the best fit model for the concatenated data set.

ML trees were constructed using PhyML version 3 [39]. The GTR model was used and the number of rate categories was set to 6. Proportion of invariable sites and gamma shape parameter were 0.92 and 1.92 respectively (estimated by MrMODELTEST). The robustness of lineages in the ML tree was tested by the bootstrap test with 100 resamplings. Bayesian inference (BI) was conducted in “partitioned by gene” using MrBayes 3.1.2 [40]. The concatenated sequence data was divided into three different partitions corresponding to the three mitochondrial regions and applied the best fit model for each partition. The model parameters were as following: lset applyto = (1, 3) nst = 6 rates = propinv; lset applyto = (2) nst = 2 rates = propinv. We allowed the overall rate to vary between partitions by setting the priors <ratepr = variable> and model parameters (e.g. gamma shape, proportion of invariable sites) unlinked across partitions. Four Metropolis-coupled Markov chains Monte Carlo (one cold and three heated) were run for 15 million iterations, with trees sampled every 1000 iterations. The first three million iterations (3000 trees) were discarded (‘burn-in’ period), and the posterior probabilities were estimated for remaining sampled iterations. Two independent Bayesian runs initiated from random starting trees were performed, and the log-likelihood values, posterior probabilities and average deviations of split frequencies were checked to ascertain that the chains had reached convergence.

### Phylogeographic structure

We used unrooted networks to explore the phylogeographic structure of *A. c. talifuensis–concinnus* because many of the underlying assumptions of traditional tree-building methods (e.g. fully bifurcating trees, complete lineage sorting) are often violated in intraspecific studies [41]. The major-joining network was constructed with program NETWORK 4.5 [42]. An analysis of molecular variance was performed using ARLEQUIN 3.1.2 [43] to test the segregation of individuals according to the haplotype clusters indicated in the phylogenetic tree. Significance levels were determined by conducting nonparametric procedures 1000 times. In addition, because of the large number of samplings of lineage C, pairwise population differentiation was estimated by computing pairwise *F*_ST_. Genetic distance and pairwise number of nucleotide differences were used to calculate the pairwise *F*_ST_. A permutation test was used to calculate the probability that two populations had no genetic differentiation. For both tests, the significant level was set to 0.01.

### MDIV analysis

We used the coalescent-based program MDIV [44] to determine the divergences between major lineages. MDIV uses a Bayesian approach to simultaneously approximate the posterior distribution of three parameters: divergence time between populations (*T = t_div_*/2*N_e_*), the migration rate between populations since divergence (*M* = 2*N_e_m*), and the population parameter theta (θ = 2*N_e_*µ, where µ is the per locus mutation rate). The program was first run using default search settings and priors (for theta and divergence time). Following this data exploration, we set our prior for *T* and M to 5. Analyses were run for 5 million generations following a burn-in period of 500,000 generations, and repeated three times to ensure convergence upon the same posterior distributions for each of the parameter estimates. Because direct rate estimates for the concentrated sequence were not available, estimates of *T* were converted to real time assuming a neutral mutation rate of 1.05×10^−8^ substitutions/site/year according to the recent studies [45], [46]. These rates were multiplied by the number of sequenced nucleotides to obtain our estimate of µ for the conversions. Divergence time estimates are intended only to give a general idea of the timescale of diversification; as with any such molecular clock estimates, they should be interpreted with caution. We used the formula 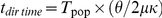
estimate divergence times.

### Historical demography

Three commonly used methods were applied to explore the demographical history of *A. c. talifuensis–concinnus*. First, statistical tests designed to assess whether nucleotide polymorphisms deviated from expectations under neutral theory [Tajima's D [47] and Fu's [48] *F_S_*] were carried out in ARLEQUIN 3.1.2. Significance of D and *F_S_* values were determined using 1000 simulated samples to produce an expected distribution under selective neutrality and population equilibrium. The cut-off level for statistical significance was 0.05. For Fu's *F_S_*, significance at the 0.05 level was indicated when P values were <0.02 [43].

Second, the program ARLEQUIN 3.1.2 was used to compare the observed frequency distribution of pairwise nucleotide differences among haplotypes with that expected from a population under expansion (Mismatch distribution analysis). For populations which have undergone historical expansions, plots of the distribution of pairwise differences among haplotypes are expected to be unimodal, whereas populations in equilibrium generally show multimodal distributions [49]. Parametric bootstrapping was used to test the goodness-of-fit between the observed mismatch and sudden expansion model, and to obtain confidence intervals [50]. The timing of population expansion was estimated by the mode (*τ*) of the mismatch distribution expressed in units of mutational time as *t = τ*/2*u* [51], where *t* is the expansion time in number of generations and *u* is the mutation rate per generation for the whole sequence.

Third, past population dynamics were examined using Bayesian skyline plots (BSP) in the program BEAST 1.4.8 [52]. This genealogical method utilizes MCMC sampling of sequence data to estimate a posterior distribution of effective population size through time and does not require a specified demographic model (e.g. constant size, exponential growth, logistic growth, or expansive growth) prior to the analysis. Bayesian skyline analyses were run under the conditions of the SRD06 model (four gamma categories), which has been found to provide a better fit for protein-coding nucleotide data. The mutation rate was the same as that used in the MDIV analyses. The number of grouped intervals (m) was set to five for lineages A and B because of the small number of sequences. For lineage C, m was set to ten. Five million iterations of the MCMC chains were run and sampled every 1000 iterations with the first 500,000 chains discarded as burnin. Bayesian skyline plots were generated with TRACER version 1.2.1 [52].

### Ecological niche models

We used algorithms of MAXENT [53]–[54] and DesktopGARP implementation of GARP with the best subsets provided by a free and open source software package OPENMODELLER [55]–[56] to predict the distribution of target species during three periods; present, LGM (0.021–0.018 Ma) and LIG (0.14–0.12 Ma). Nineteen available environment data layers generated from CCSM paleo-climatic model [57] during three periods were downloaded from the WorldClim dataset (http://www.worldclim.org/) [58]. These data are high-resolution [58] and have been applied to investigate Pleistocene climatic effects on an East Asian bat, the greater horseshoe bat *Rhinolophus ferrumequinum* [59].The data layers were 2.5′ spatial resolution for the present and LGM. The 30″ spatial resolution layers for LIG were subsequently resampled to a spatial resolution of 2.5' using ARC GIS 9.3. These environmental variables reflected annual trends for temperature and precipitation, bioclimatic seasonality, extreme conditions or potential limiting factors. ENMs were built according to current environmental factors, and then projected to three periods. All sample points were used as the model input.

For MAXENT, we used the quadratic features and set the maximum iterations to 2000, replicates to 10. Eighty percent of the localities were randomly selected to train the model and the remaining 20% to test the model performance. The fade-by-clamp option was selected to remove heavily clamped pixels from the final prediction. Other parameters were used default values. For GARP, We changed the total run to 100, max generations to 900 and training proportion to 80. The training data was divided into true training data (for model rule development) and intrinsic testing data (for rule-testing intrinsic to GARP processing), and the remaining 20% data was set aside for an independent test of model quality. The ‘best subset’ was generated according to the soft omission threshold of 20% and the commission threshold of 50%.

The value of AUC (Area under curves), sensitivity and omission error were used to assess the model's performance [60]–[61]. We selected the Arc/Info ASC II Grid as output format, which can be easily handled with GIS software. The output maps were imported to ARC GIS 9.3 to produce final maps. Different map colors corresponded to different fitting indices in the potential distribution maps. The two algorithms generated the similar potential suitable distribution for each period but were different in range and location of the most suitable habitat. As the GARP modelling also showed the values of sensitivity (1) and omission error (0) [61], we described the change of species distribution mainly based on the predictions of GARP but drew the conclusions by adopting a conservative approach by including areas predicted as ‘suitable’ or “unsuitable” by both models [59], [62].

## Results

### Sequence variation and genetic diversity

The combined length of the obtained sequences (3192 bp) contained 336 polymorphic sites, 235 of which were parsimony informative. These polymorphic sites defined 139 unique haplotypes, 112 of which were observed in a single bird. Two widespread haplotypes were observed and shared by 15 individuals from different sampling sites. For sample sizes larger than four, haplotype diversity values were nearly maximal: from 0.8333 to 1; nucleotide diversity values were between 0.052% and 0.378% (Table S1). Phylogenetic relationship inference identified three lineages (Marked with A, B, C) and the nucleotide diversity for them is 0.326%, 0.145% and 0.179% respectively. Samples collected at site LJ included the haplotypes of lineage A and lineage B, while those collected at PZH included the haplotypes of lineage B and lineage C. These populations all show extremely high values of nucleotide diversity (values not shown).

### Phylogenetic relationships

**Figure 2:**
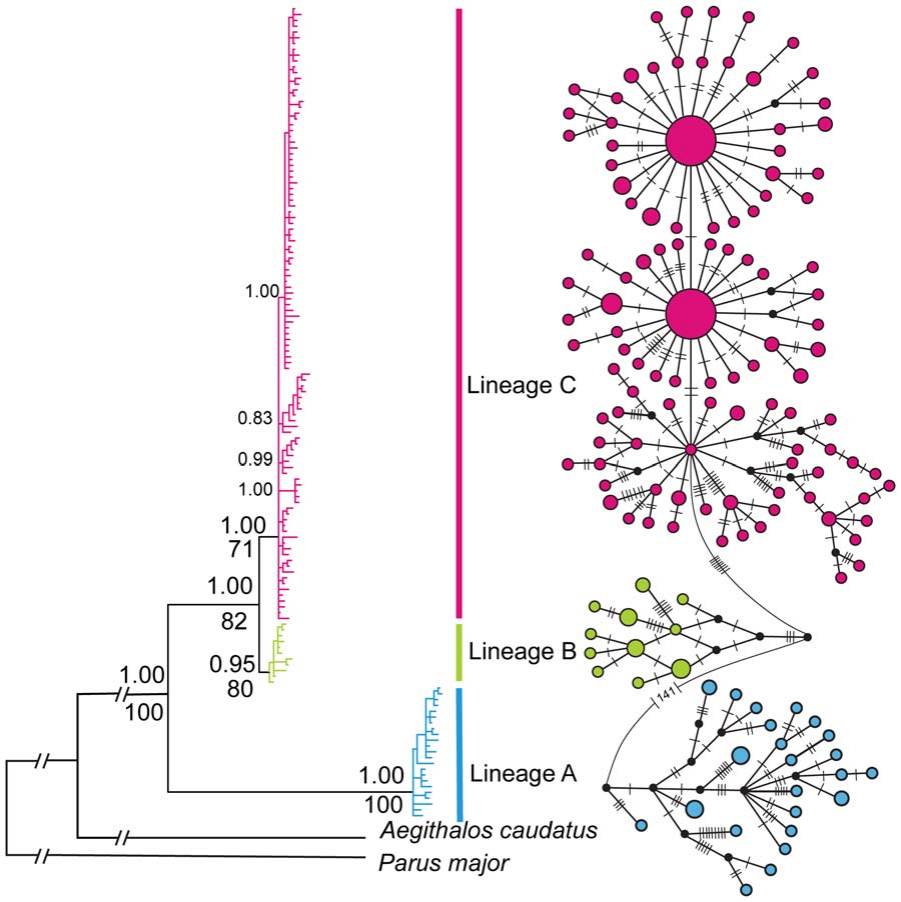
Phylogenetic relationships among haplotypes. The inferred phylogenetic tree and the Major-joining network based on the combined COI, *cyt b* and ND2 genes. Numbers above tree branches are the posterior probabilities for the BI and bootstrap values for the ML below. Dashes and numbers in the network represent the corresponding mutation steps.

The Maximum likelihood and Bayesian phylogenetic inferences both divided our data into three major clades with strong support for both the lineages themselves and the relationships between them (Fig. 2). The primary geographical lineages identified are as follows:

Clade A: A lineage comprising sampling areas spanning Yunnan Province.

Clade B: This lineage is restricted to the region between northern Yunnan Province (Lijiang) and southwest Sichuan Province (Panzhihua, Yanbian and Miyi counties).

Clade C: The largest lineage which is distributed from the western border of Guizhou and Guangxi, across the wide areas of central China, to the eastern seaboard.

All three lineages are strongly supported by posterior probabilities (PP>95%) and bootstrap values (BV>70). The relationships among these lineages are also supported at greater than 95% PP and 70 BV. Lineage B and C are inferred to be sister taxa, whereas lineage A is a sister to B and C. Several well supported clades (PP>95%) are apparent within lineage C. In terms of the current taxonomy, subspecies *A. c. talifuensis* includes lineages A and B, whereas lineage C, with the exception of samples collected at site PZH which includes individuals of both lineages B and C, exhibits strong concordance between the geographical mtDNA lineage and the currently described nominate subspecies, *A. c. concinnus*.

### Phylogeographic structure

The constructed major-joining network revealed three major clusters which were congruent with the three clades in the phylogenetic tree (Fig. 2). Within the cluster C, star-like clades indicative of recent population expansions are apparent (Fig. 2). AMOVA analysis showed that most genetic variation (93.93%) was among groups, with that within populations accounting for just 5.17% of the total. Genetic variation among populations within groups was just 0.9% (Table 1). Comparison of the populations within lineage C indicates that birds from the westernmost sites (PZH and JZS) were significantly different from those at other sites and that gene flow between these and other lineage C sites would be almost impossible because of the number of migrants per generation *N_m_*<1.

**Table 1:** Hierarchical analyses of molecular variance of the *A. c. talifuensis–concinnus.*

Source of variation	d. f.	Sum of squares	Variance of components	Percentage of variance (%)	Ф Statistics (P-value)
Among groups	2	3894.432	49.257	93.93	Ф_CT_ = 0.9393*
Among populations within groups	33	173.899	0.471	0.9	Ф_SC_ = 0.1479*
Within populations	167	455.561	2.711	5.17	Ф_ST_ = 0.9482*
Total	202	4523.892	52.440		

(*P<0.001).

### Divergent times and migration rates among major clades

The estimated divergence time between lineage A and lineage B–C was 1.79 (95% CI: 0.84 – 2.68) coalescence units. Using our estimated mutation rate to convert the MDIV estimate to real time, we estimate a divergence time of approximately 2.4 Ma (95% CI: 4.29 – 0.88 Ma). Lineages B and C were estimated to diverge at 0.29 (95% CI: 0.25 – 0.56) coalescent units, which converted to real time corresponds to 0.32 Ma (95% CI: 0.76 – 0.21 Ma). The migration rate between lineage A and lineage B–C is near zero (95% CI: 0 – 0.44). Estimation of the migration rate between lineage B and lineage C suggests a rather restricted gene flow M = 0.12 (95% CI: 0.03 – 0.91).

### Historical population demography

**Figure 3:**
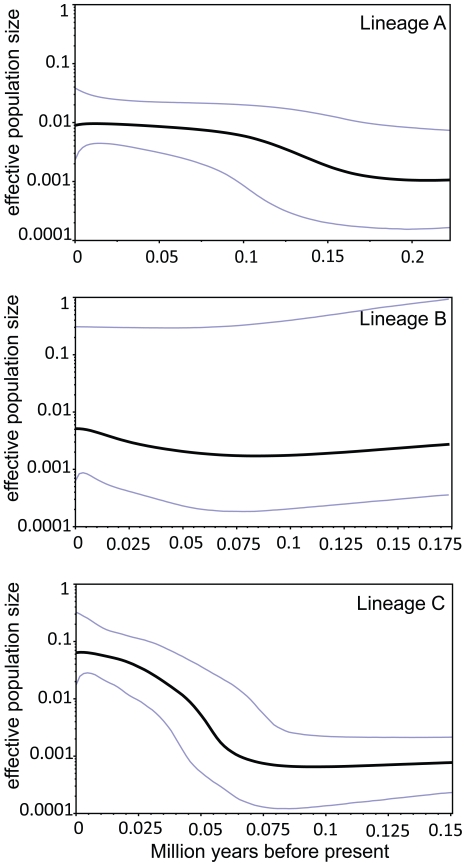
Bayesian skyline plots (BSPs). The x-axis measures time in millions of years and the y-axis is the scaled effective population size. Dark lines represent media inferred *Ne,* blue lines mark the 95% highest probability density (HPD) intervals in all panels for lineage A (a), for lineage B (b), and lineage C (c).

Tajima's D and Fu's *Fs* statistics were negative for all three lineages. However, only the statistics values for lineages A and C were significant. For mismatch analyses, lineages A and B showed a multimodal distribution profile, but the statistical mismatch distribution test statistics (SSD and rg) were not significant. Plots of the distribution of pairwise differences for lineage C are unimodal (Table 2). This distribution type, together with the very small and not statistically significant mismatch distribution test statistics, suggests that this lineage has undergone recent population expansions. Using the same mutation rate for the whole sequence as it was in the MDIV analysis, the expansion time for lineage C was estimated at about 0.07 Ma. Bayesian skyline plots showing the estimated changes in median *N_ef_* over time for the three lineages were concordant with the results from the mismatch distribution. Fig. 3 shows that lineage A experienced a long period of relative population stability before undergoing an increase about 0.2−0.1 Ma after which it again remained stable until the present. Lineage B seems to have experienced a population reduction followed by a recovery at about 0.05 Ma. However, the 95% highest probability density (HPD) intervals on this estimate are very large. Lineage C appears to have undergone population expansions since about 0.075 Ma, after which it entered a period of relative population stability.

**Table 2:** Nucleotide polymorphism and results of neutrality tests and mismatch distribution analysis for lineages.

	lineage	A	B	C	Total
Sample sizes	*n*	29	19	155	203
Nucleotide polymorphism	S	69	21	167	336
	Nhap	23	11	105	139
	Hd	0.98	0.924	0.98	0.987
	*π*	0.326%	0.145%	0.179%	1.401%
Neutrality tests	D (P value)	-1.54 (0.03)	-0.892 (0.19)	-2.598 (0.00)	/
	*Fs* (P value)	-7.928 (0.005)	-2.256 (0.116)	-25.091 (0.00)	/
Mismatch analysis	Distribution type	multimodal	multimodal	unimodal	/
	SSD (P value)	0.008 (0.29)	0.028 (0.36)	0.001 (0.74)	/
	Rg (P value)	0.016 (0.25)	0.050 (0.57)	0.005 (0.88)	/

### Ecological niche models

**Figure 4:**
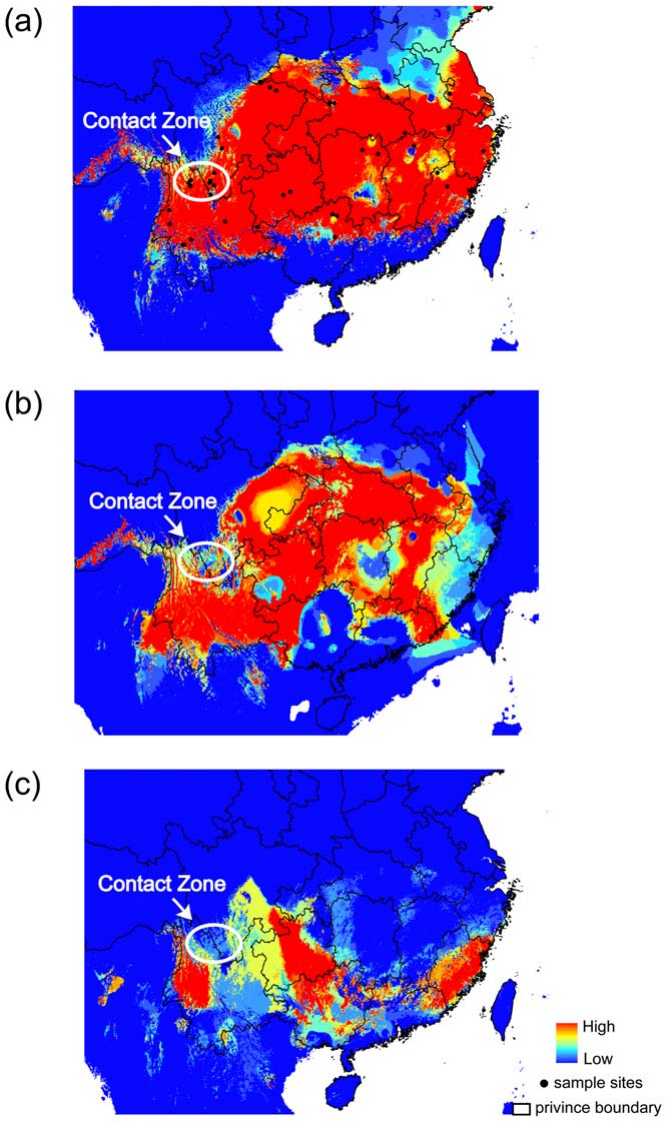
Ecological niche models predicted distributions using GARP during three periods. Different colors corresponded to different fitting indices with low in blue and high in red for the current distribution (a), LGM distribution (b) and LIG distribution (c).

The developed ecological niche models distributional predictions for present, LGM and LIG are given in Fig. 4 and Figure S1. A good and strong fit between the model and data is suggested by the high values of AUC (0.95 for GARP and 0.947 for MAXENT respectively), and the high coincidence between the projected distributions and collection records.

All modeled distributions suggest that there has been suitable habitat for the black-throated tit in Yunnan since the LIG. The species' predicted present geographic range indicates large areas of suitable habitat in south-western and southern China (Fig. 4a and Figure S1a). Modelling suggests that this bird is absent from Hainan Island, southern Guangxi and southern Guangdong provinces. This is confirmed by our field and other observational records (http://birdtalker.net/index.asp). However, GARP model reconstructions fail to project Taiwan as a suitable habit, possibly because of a lack of data. The ENMs suggest that the black-throated tit had distribution similar to the present during LGM (Fig. 4b and Figure S1b). The species' entire potential range and preferred habitat were only slightly reduced. However, projections for LIG are unexpected. The modeled distributions indicate a substantial reduction in range, with almost no suitable habitats predicted in the center and north of the current distribution (Fig. 4c and Figure S1c). During this period the species' range appears to have been restricted to Yunnan, southern Sichuan, Guizhou, Guangxi, Guangdong and Fujian provinces.

## Discussion

### MtDNA lineages diversification and the demography history

Previous studies suggested that there was no genetic divergence within *A. c. concinnus*-*talifuensis* [28]–[29]. In contrast, we found significant genetic divergence in this group, despite its current continuous distribution. Both phylogenetic inferences and haplotype network analyses revealed the existence of three distinct evolutionary lineages in *A. c. concinnus*-*talifuensis*. Node support for each lineage is high and missteps in haplotype network connections are large, especially between lineages A and B–C (up to 141 steps). Furthermore, analysis of molecular variance partitioned data into three phylogroups with clearly defined geographical population structures. We noticed that the sampling sites of specimens for *A. c. talifuensis* analyzed by the previous studies were located in the area of lineage C. Thus, we compared the genetic distance (cyt b, p-distance) for a specimen of lineage A (*A. c. talifuensis*) with other two subspecies specimens (*A. c. manipurensis* and *A. c. iredalei*) which are available at GenBank (Accessions: HM185382, GU244428, GU244426). The genetic distance was 4.9% between *A. c. talifuensis* and *A. c. manipurensis* and 5.1% between *A. c. talifuensis* and *A. c. iredalei*. These results suggest that the black-throated tit displays high genetic differentiation and zones of lineage endemism even within the Chinese part of its range.

The significant values for Tajima's D and Fu's *Fs* statistical tests and the unimodal mismatch distribution suggest that lineage C has undergone a past population expansion. For lineage A and lineage B, however, values of statistical tests (significantly negative or negative) and mismatch distributions (multimodal but not significant values of goodness-of-fit tests) were not consistent with each other well. The coalescent-based method, which incorporates information from the genealogical tree structure of DNA sequences, seemed to be a more effective way of estimating demographic processes. Bayesian skyline plots indicate different population dynamics among lineages; a relatively constant population size in lineage A, a past population reduction and recent slight increase in lineage B, and successive population expansions in lineage C. Although BSP confidence intervals for lineage B were large, they are all supported by the ENMs. ENMs indicate that the amount of suitable habitat available was constant for lineage A, very limited range for lineage B during both the LIG and LGM, and a reduced distribution for lineage C during LIG.

### Climatic changes and lineage divergence

The genetic distances (cyt b, p-distance) between lineages (of 4.8% between lineages A and B and 4.9% between lineages A and C) indicate relatively clear genetic separation between these lineages. However, the reasons for this are not clear and there is no obvious geographic barrier to dispersal in the current distribution. Marked genetic divergence within continuous habitat generally could be due to a cryptic barrier, or be a relic of a now absent historical barrier. Recently, the uplift of the Tibetan plateau has been hypothesized to induce phylogeographic breaks within or among species both on and outside the plateau in Southeast Asia [23], [63]. The uplift of Tibetan plateau had profound effects on the geological environment of the Plateau and adjacent areas [21] and may have promoted the habitat fragmentation of species. However, reconstructing the paleo-vegetation in western Yunnan suggested that Late Pliocene vegetational patterns were similar to those of the present and that the Hengduan Mountains had approached their highest elevation before the Late Pliocene [64]. These findings suggest that the habitat of the black-throated tit may already have existed in the Late Pliocene. Considering that lineage A and the common ancestor of lineages B and C diverged at the estimation of about 2.4 Ma (95% CI: 4.29 − 0.88 Ma), and lineages A and B currently co-distributed in the Yulong Snow-Mountain of the Hengduan Mountains, the genetic split of black-throated tit was unlikely to be shaped by the uplift of Tibetan plateau.

Alternatively, our molecular dating and ENMs reconstructions strongly suggest that Pleistocene climatic oscillations were more likely the reasons for the divergence between lineage A and lineages B–C. Our ENMs reconstructed for LIG and LGM, especially for LIG, show that the present contact zone among those lineages was relatively unsuitable. The predictive fragmented habitats would indicate the major refuges for the ancestors of those extant lineages. Therefore, climatic oscillations had real impact on the distribution of the black-throated tit. As the starting point of diversification is given right at the Pliocene/Pleistocene with 2.4 Ma, this separation was very likely under some Pleistocene impact at least with respect to the first cooling event. Given the fact that the ENMs simulations represent just the final phase of a series of repeated glacial cycles and fragmentation events, there were surely multiple effects of population decrease and expansion during the total time frame of lineage divergence. Thus, it was likely that climatic oscillations had caused the divergence between lineages A and B–C.

Southeast Asia had experienced several climatic oscillations during the last 0.4 million years [65]. It has been suggested that these climatic oscillations were major factors in the isolation of lineages in many species [23], [24], [66]. For instance, it has been hypothesized that the expansion of the Fagaceae forest type following climatic cooling about 0.35 Ma restricted the various lineages of the white-browed piculet (*Sasia ochracea*) to mountainous refugia resulting in increased genetic differentiation [66]. Similarly, since the estimated divergence time between lineages B and C was 0.32 Ma (95% CI: 0.76 − 0.23 Ma), we think it likely that climate-driven habitat fragmentation was the cause of divergence between lineage B and lineage C.

### The LGM effect on species' distribution

Last Glacial Maximum of Pleistocene climatic oscillations (0.021−0.018 Ma) had a profound impact on species' distribution and population sizes, e.g. in the heavy ice sheets covering northern regions of Europe and North America [5], [7], [67]. In contrast, our results show that LGM had little impact on the geographic distribution of *A. c. talifuensis–concinnus.* This conclusion is supported by both the ENMs and phylogeographic analyses. For instance, the ENMs suggest that there has been suitable environment for lineage A in Yunnan since the LIG. This result, combined with the constant population suggested by Bayesian skyline analyses, indicates that LGM may have had little impacts on this lineage. For lineage C, Bayesian skyline and mismatch analyses detect a strong signal of a recent population expansion across most of this lineage's current range. However, because the ENMs suggest that a similar geographic range as the present during the LGM and much of its LGM habitat was uninhabitable during the LIG, and the genetic data suggest that the range of this lineage expanded before the LGM (about 0.07 Ma), we think that LGM effects were limited. This is also true for lineage B, as our result showed that this lineage has a more restricted distribution during LGM than it does at present but more suitable habitat than it had during LIG. However, the significant genetic divergence between the western (PZH and JZS) and other populations within lineage C suggests that small refugia may have existed. Similar results were found in a phylogeographic study of the canopy tree *Eurycorymbus cavaleriei* [68].

In fact, our reconstructions of LGM distribution are much congruent with previous phylogeographic studies [23]–[25], [59], [69], [70]. These studies suggested the existence of multiple refugia, but species all showed population expansion before the LGM. Our ENMs and molecular data support that East China was not subjected to significant glacial advances [71], at least with respect to the period of LGM. Unexpectedly, our ENMs results provide a distinct perspective for the climatic impact of LIG. However, this conclusion requires evaluation in other taxa with different life-history traits and biology.

### Contact zone and the refuge theory

Contact zones generally refer to the sympatric areas of closely related species which have their own separated primary distributions. Contact zones are important to understand the process of speciation, dispersal and vicariance. It is known that many closely related species pairs of birds are distributed in parapatry or allopatry with narrow contact zones [72]–[74]. In this study, we found a narrowly circumscribed intraspecific contact zone whereby lineage B was parapatrically distributed with each of the two related lineages in LJ and PZH respectively.

A plethora of hypotheses have been suggested to explain the cause of contact zones, such as the refuge, river refuge and disturbance-vicariance hypotheses [73], [75]. Among these, the refuge theory of Haffer [73], [75] argued that the contractions and expansions of species range influenced by the climatic fluctuation would establish secondary contact zones. Our ENMs reconstructions support this theory. As can be seen in Fig. 4, the contact zone was projected as unsuitable for the black-throated tit during LIG and LGM though the present ENMs showed that the habit was suitable. The present parapatry of these lineages could be the result of the recent expansions in the range of lineages B and C indicated by the BSP analysis into the present suitable environment. In addition, several species or species group have differentiated in a similar way of the black-throated tit, such as *Seicercus affinis* [76], *Cettia flavolivacea* [77], *Certhia discolor* [78], and species group of *Phylloscopus cantator* and *P. ricketti* [79], especially for a subspecies group between *Certhia familiaris bianchii* and *C. f. khamemsis* [78]. Although, the contact zone hotspot of birds appears to be located in Middle East [73]–[74], these findings indicate that areas between LJ and PZH may also be a contact zone hotspot whereby this range has been considered to play an important role in avian endemism and speciation in China [19].

## Supporting Information

Table S1 **Coordinates, haplotype diversity and nucleotide diversity of each sampled population.**
(DOC)

Figure S1 **Ecological niche models predicted distributions using Maxent for the current (a), LGM (b) and LIG (c).**
(TIF)
