# Supplementary material for: Profound Climatic Effects on Two East Asian Black-Throated Tits (Ave: Aegithalidae), Revealed by Ecological Niche Models and Phylogeographic Analysis
Source: PLoS One. 2011 Dec 16;6(12):e29329. doi: 10.1371/journal.pone.0029329 (PMC3241714; doi:10.1371/journal.pone.0029329)
Supplement: Table S1 — Coordinates, haplotype diversity and nucleotide diversity of each sampled population. (DOC) [file pone.0029329.s001.doc]

**S 1**

Coordinates, haplotype diversity and nucleotide diversity of each sampled population.

| Phylogroup | Sample sites | Longitude(E)/Latitude(N) | n | H | h | π(%) | Genbank accession No. | | |
| --- | --- | --- | --- | --- | --- | --- | --- | --- | --- |
|  |  |  |  |  |  |  | COI | *cyt b* | ND2 |
| Lineage A | Kungming (KM) | 102.62/24.97 | 3 | 3 | 1 | 0.251 | HQ605249, 53, 63 | HQ605452, 56, 66 | HQ605655, 59, 69 |
|  | Dahaoping (DHP) | 98.73/25.59 | 9 | 7 | 0.9166 | 0.378 | HQ605250-52  HQ605267-72 | HQ605453-55  HQ605470-75 | HQ605673-78,  HQ605656-58 |
|  | Baoshan (BS) | 98.77/24.82 | 2 | 1 | 0 | 0 | HQ605257-58 | HQ605460-61 | HQ605663-64 |
|  | Yuanxian (LC) | 100.27/23.99 | 1 | 1 | / | / | HQ605254 | HQ605457 | HQ605660 |
|  | Gengma (LC) | 99.70/23.69 | 1 | 1 | / | / | HQ605256 | HQ605459 | HQ605662 |
|  | Shuangjiang (LC) | 99.82/23.70 | 1 | 1 | / | / | HQ605255 | HQ605458 | HQ605661 |
|  | Lijiang^a^ (LJ) | 100.07/27.10 | 12 | 9 | 0.9393 | 0.239 | HQ605260-62, 64, 66  HQ605278, 79, 81, 84-86, 88 | HQ605463-65, 67, 69  HQ605481, 82  84, 87-89, 91 | HQ605666-68, 70, 72  HQ605684, 85, 87, 90-92, 94 |
| Lineage B | Lijiang^a^ (LJ) | 100.07/27.10 | 6 | 3 | 0.7333 | 0.188 | HQ605280, 82-83, 87  HQ605259, 65 | HQ605483, 85-86, 90  HQ605462, 68 | HQ605686, 88-89, 93  HQ605665, 71 |
|  | Miyi^b^ (PZH) | 101.83/26.96 | 6 | 5 | 0.9333 | 0.052 | HQ605218  HQ605206, 08  HQ605210-12 | HQ605421  HQ605409, 11  HQ605413-15 | HQ605624  HQ605612, 14  HQ605616-18 |
|  | Yanbian^b^ (PZH) | 101.59/27.23 | 5 | 4 | 0.9000 | 0.069 | HQ605190  HQ605201  HQ605213-15 | HQ605393  HQ605404  HQ605416-18 | HQ605596  HQ605607  HQ605619-21 |
|  | Panzhihua^b^ (PZH) | 101.60/26.45 | 1 | 1 | / | / | HQ605216 | HQ605419 | HQ605622 |
|  | Yanyuan (PZH) | 101.84/27.64 | 1 | 1 | / | / | HQ605191 | HQ605394 | HQ605597 |
| Lineage C | Panzhihua^b^ (PZH) | 101.60/26.45 | 1 | 1 | / | / | HQ605217 | HQ605420 | HQ605623 |
|  | Yanbian^b^ (PZH) | 101.59/27.23 | 1 | 1 | / | / | HQ605204 | HQ605407 | HQ605610 |
|  | Miyi^b^ (PZH) | 101.83/26.96 | 3 | 3 | 1 | 0.292 | HQ605205, 07, 09 | HQ605408, 10, 12 | HQ605611, 13, 15 |
|  | Jixi (JX) | 118.54/30.20 | 14 | 12 | 0.9780 | 0.214 | HQ605086-99 | HQ605289-302 | HQ605492-505 |
|  | Jianou (JO) | 118.14/26.90 | 10 | 8 | 0.9272 | 0.107 | HQ605113-15  HQ605117-23 | HQ605316-18  HQ605320-26 | HQ605519-21  HQ605523-29 |
|  | Dongzai (DZ) | 114.30/31.86 | 12 | 10 | 0.9697 | 0.219 | HQ605100-11 | HQ605303-14 | HQ605506-17 |
|  | Guiyang (GZ) | 106.67/26.42 | 19 | 18 | 0.9941 | 0.171 | HQ605146-55  HQ605157-59  HQ605161-66 | HQ605349-58  HQ605360-62  HQ605364-69 | HQ605552-61  HQ605563-65  HQ605567-72 |
|  | Guilin (GL) | 110.06/25.37 | 14 | 11 | 0.9670 | 0.149 | HQ605128-36  HQ605140-44 | HQ605331-39  HQ605343-47 | HQ605534-42  HQ605546-50 |
|  | Nanjing (NJ) | 118.79/32.07 | 10 | 7 | 0.9333 | 0.165 | HQ605180-89 | HQ605383-92 | HQ605586-95 |
|  | Shengnongjia (SNJ) | 110.14/31.57 | 15 | 13 | 0.9809 | 0.127 | HQ605219-33 | HQ605422-36 | HQ605625-39 |
|  | Haoping (HP) | 107.78/34.00 | 15 | 12 | 0.9428 | 0.111 | HQ605234-48 | HQ605437-51 | HQ605640-54 |
|  | Guangyuan (GY) | 105.84/32.42 | 8 | 8 | 1 | 0.148 | HQ605195-200  HQ605202-03 | HQ605398-403  HQ605405-06 | HQ605601-06  HQ605608-09 |
|  | Xianju (XJ) | 120.85/28.85 | 5 | 5 | 1 | 0.263 | HQ605273-77 | HQ605476-80 | HQ605679-83 |
|  | Wenxian (WX) | 105.21/32.70 | 4 | 4 | 1 | 0.094 | HQ605124-27 | HQ605327-30 | HQ605530-33 |
|  | Pingjiang (PJ) | 112.30/28.92 | 4 | 3 | 0.8333 | 0.110 | HQ605169-72 | HQ605372-75 | HQ605575-78 |
|  | Yuanjiang (YJ) | 113.39/28.69 | 4 | 3 | 0.8333 | 0.084 | HQ605173-76 | HQ605376-79 | HQ605579-82 |
|  | Chishui(CS) | 105.70/28.59 | 3 | 3 | 1 | 0.063 | HQ605145, 56, 60 | HQ605348, 59, 63 | HQ605551, 62, 66 |
|  | Wuxiu (WuX) | 115.97/29.13 | 3 | 3 | 1 | 0.188 | HQ605177-79 | HQ605380-82 | HQ605583-85 |
|  | Jianyang (JY) | 117.50/27.65 | 2 | 2 | 1 | 0.188 | HQ605112, 16 | HQ605315, 19 | HQ605518, 22 |
|  | Yangxin (YX) | 115.38/29.87 | 2 | 1 | 0 | 0 | HQ605167-68 | HQ605370-71 | HQ605573-74 |
|  | Jinzhongshan (JZS) | 104.87/24.66 | 2 | 2 | 1 | 0.439 | HQ605138-39 | HQ605341-42 | HQ605544-45 |
|  | Moxi (MX) | 102.12/29.64 | 2 | 2 | 1 | 0.157 | HQ605192-93 | HQ605395-96 | HQ605598-99 |
|  | Liziping (LZP)) | 102.38/29.01 | 1 | 1 | / | / | HQ605194 | HQ605397 | HQ605600 |
|  | Jinxiu (JiX) | 110.19/24.12 | 1 | 1 | / | / | HQ605137 | HQ605340 | HQ605543 |
|  |  |  |  |  |  |  |  |  |  |
|  | Total |  | 203 | 139 | 0.934 | 1.311 |  |  |  |

1. Sympatric populations of Lineage A and Lineage B.
2. Sympatric populations of Lineage B and Lineage C.
