# Supplementary figures and images for: Profound Climatic Effects on Two East Asian Black-Throated Tits (Ave: Aegithalidae), Revealed by Ecological Niche Models and Phylogeographic Analysis
Source: PLoS One. 2011 Dec 16;6(12):e29329. doi: 10.1371/journal.pone.0029329 (PMC3241714; doi:10.1371/journal.pone.0029329)

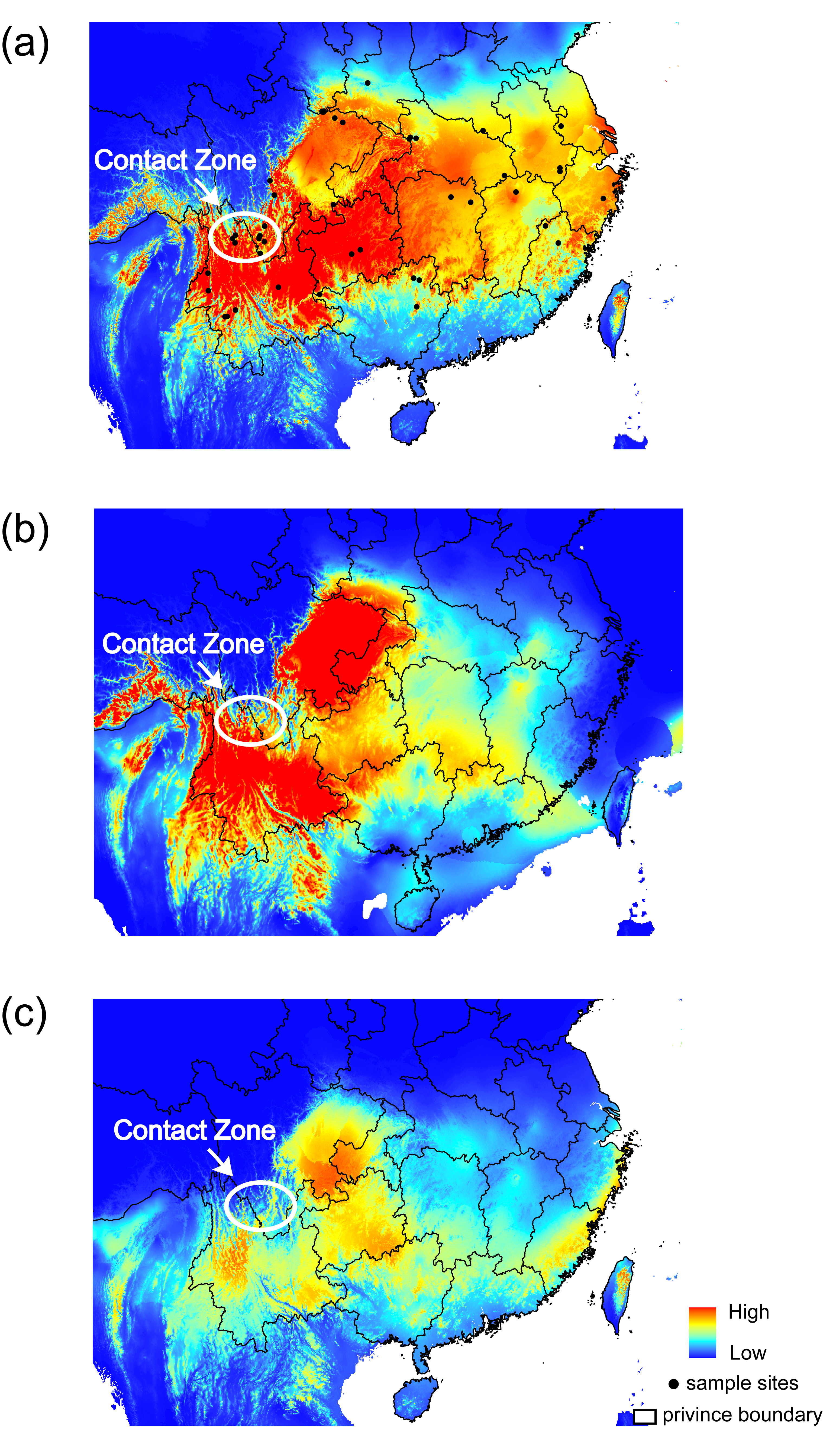

Supplement: Figure S1 — Ecological niche models predicted distributions using Maxent for the current (a), LGM (b) and LIG (c). (TIF) [file pone.0029329.s002.tif]
